# Histopathologic patterns of cutaneous malignancies in individuals with oculocutaneous albinism in Anambra state, Nigeria: a paradigm swing?

**DOI:** 10.3332/ecancer.2020.1013

**Published:** 2020-02-20

**Authors:** Nkechi Anne Enechukwu, Gabriel Olabiyi Ogun, Ogochukwu Ifeanyi Ezejiofor, Titus Osita Chukwuanukwu, Joseph Yaria, Adekunle Olufemi George, Adebola Olufunmilayo Ogunbiyi

**Affiliations:** 1 Department of Internal Medicine, Nnamdi Azikiwe University, Awka, Nnewi Campus, Anambra State, Nigeria; 2 Department of Pathology, University College Hospital, Ibadan, Oyo State, Nigeria; 3 Department of Surgery, Nnamdi Azikiwe University, Awka, Nnewi Campus, Anambra State, Nigeria; 4 Department of Medicine, University College Hospital, Ibadan, Oyo State, Nigeria

**Keywords:** oculocutaneous albinism, cutaneous malignancies, dermatopathology, dermoscopy

## Abstract

**Background:**

A high proportion of skin cancers in Nigeria occur in Individuals with oculocutaneous albinism (OCA). A reduction or absence of melanin, a skin pigment with photoprotective properties, makes them susceptible to skin malignancies such as squamous cell carcinomas (SCCs), basal cell carcinomas (BCCs) and rarely melanomas. Globally, BCCs are the commonest cutaneous malignancies among Caucasians and in fair-skinned Africans. This has been attributed to the greater effect of melanin in protecting against UV damage in the basal layer of the epidermis. Older retrospective studies on African albinos suggested that SCCs accounted for a higher prevalence of skin cancers in albinos, followed by BCCs. Melanoma has been consistently documented to be rare in all of these reports. Recent reports however noted BCCs to occur at an increasing frequency, suggesting a higher frequency than previously documented. These conflicting reports reflect the need to re-explore the pattern of cutaneous malignancies in albinos in order to reconcile the role of pigmentation, UV exposure and the variance between the frequencies of the different keratinocyte skin cancers among extreme skin phenotypes. This study explores the pattern of cutaneous malignancies seen in albinos in South East Nigeria.

**Objective:**

To determine the pattern of cutaneous malignancies among albinos in Anambra state, Nigeria.

**Materials and methods:**

A cross-sectional study conducted in Anambra State, Nigeria. Ninety albinos from the Albino foundation Anambra state were recruited. Malignant dermatoses were characterized clinically and confirmed by histology. Fifty-eight lesions from 30 albinos were biopsied to determine the presence of malignancy.

**Results:**

Skin cancers were seen in 20.98% of all participants and in 18 (60%) of all the albinos who had skin biopsy. The SCC/BCC ratio was 1.0: 2.3. There was no cutaneous melanoma.

**Conclusion:**

Contrary to previous reports, it would appear that the pattern of cutaneous malignancies in albinos shows the same trend as that seen in Caucasians and fair-skinned Africans.

## Introduction

Although several studies in predominantly dark-skinned Africans show a preponderance of squamous cell carcinoma (SCCs) [1–5], studies in Africans with lighter skin phenotypes show a pattern similar to that in whites [6–10]. The relatively uncommon incidence of basal cell carcinomas (BCCs) in black Africans has been attributed to the protective effect of increased pigmentation. This is supported by the inverse relationship between melanin quantity and the occurrence of BCCs [11, 12]. Thus, when melanin content is low, BCCs tend to occur. Studies on cutaneous photobiology show that melanin exerts the highest degree of protection against UV damage in the basal layer [13]. This suggests that the absence of melanin in the skin will predispose an individual to develop BCCs which originate from basaloid cells in the basal layer. This is a possible explanation for the greater incidence of BCCs in Caucasians and the fair-skinned among others [13].

Individuals with oculocutaneous albinism have low or no melanin with their skin phenotype corresponding to Fitzpatricks type I/II. It is therefore expected that BCCs should be more common and will follow the pattern of skin cancers seen in lighter complexioned Africans and in Caucasians among this population due to the reduction or absence of melanin associated with albinism. However, data on the pattern of skin cancers among albinos in Africa are inconsistent. Although most of the older retrospective studies have suggested that SCCs accounted for a higher prevalence of the skin cancers found in albinos [1–5, 14], with melanomas reportedly rare [15, 16], recent reports have shown that BCCs are as common as SCCs in albinos [17–19]. It is also important to note that most of the reported studies were retrospective involving skin biopsies which had been submitted to the laboratories in the management of such patients. Hence the possibilities of underreporting of early BCCs or precancerous lesions cannot be ruled out.

These conflicting reports reflect the need to re-explore the pattern of cutaneous malignancies in albinos in order to reconcile the role of pigmentation, UV exposure and the variance between the frequencies of the different keratinocyte skin cancers among extreme skin phenotypes. A relatively large number of individuals with oculocutaneous albinism live in South-East Nigeria [1, 20, 21], and albinos account for a greater percentage of individuals with skin cancers in Nigeria [2, 14]. This study reports on the histopathologic pattern of cutaneous malignancies seen in a cross-section of albinos in the South East of Nigeria.

## Methodology

This was a descriptive cross-sectional study involving members of the albino foundation, Anambra state chapter. Ethical approval was obtained from the Institutions Research Board (Nnamdi Azikiwe University Teaching Hospital). Both verbal and written consents were obtained from participants. A total of 90 individuals living with albinism were recruited into the study. All consenting albinos of all ages were recruited into the study.

Participants with immunosuppression(drug-induced or from HIV, smoking history, exposure to ionizing radiation, skin lesions suggestive of Gorlin–Goltz syndrome and xeroderma pigmentosum were excluded.

**Figure 1.**
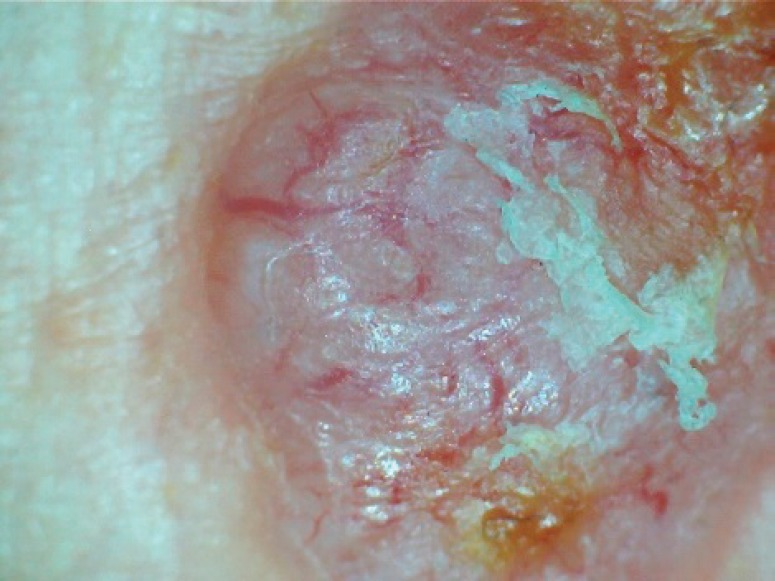
Dermoscopic picture of a BCC in an albino showing arborizing vessels and areas with ulcerations.

They had their skin examined for the presence of any dermatoses. Clinical diagnosis was made based on compatible clinical features combined with typical dermoscopic characteristics [22]. Dermoscopic features like arborizing telangiectasia, blue grey ovoid nests, shiny white blotches, ulcerations, leaf-like structures and spoke wheel-like structures were considered features of BCCs (see Figure 1); polymorphic vascular patterns, dotted and/or glomerular vascular patterns, white or yellow structureless areas, strawberry pattern and ulcerations were suggestive of SCCs, while the presence of blue white veil, polymorphous vascular patterns, regression structures (scar-like areas and ‘peppering’) and structureless areas were considered suggestive of melanoma [22]. Where the clinical and or dermoscopic diagnosis of the skin findings was in doubt or there was a diagnosis of possible premalignant or malignant dermatoses, a skin biopsy was performed after obtaining an informed consent. Tissue biopsy samples were collected in formalin containers and transferred to the histopathology laboratory for histologic diagnosis.

All data were extracted from the questionnaires and input into SPSS analytical software package version 22 after data cleaning. Baseline socio-demographic characteristics and clinical findings of the participants were reported as proportions for categorical variables and mean (SD) for continuous variables after the test of normality. Bivariate analysis was carried out using Fisher’s exact and Wilcoxon rank sum. Statistical significance was defined as when *p*-value < 0.05. Skin biopsies were also sub-analysed with Fisher’s exact and Wilcoxon rank sum used to compare demographic, clinical and sunlight associated variables between participants with malignant skin lesions and those without. Statistical significance was defined as when *p*-value < 0.05.

## Result

**Figure 2.**
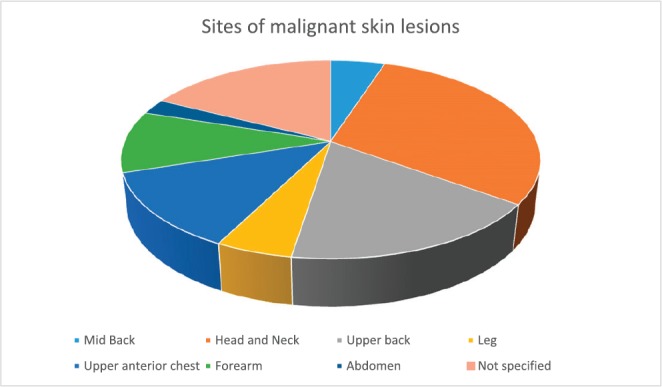
Sites of the cutaneous malignancies in the study participants.

Out of a total of 90 albinos seen, 34 (38%) had a clinical diagnosis of suspected pre-malignant (actinic keratosis) while 29 (32%) had suspected malignant skin lesions. A total of 58 skin tissue biopsy specimens were taken from 30 consenting albinos, 16 (53.3%) of which were males and 14 (46.7%) females. Albinos who had malignant lesions were older, median age 39 (IQR 33–47) years, than those who did not, median age 19 (IQR 10–33) years (*p*: 0.002). There was no difference in gender distribution between albinos with malignancies and those who did not have malignant lesions (*p*: 0.654) (Table 1). There was no difference in other socio-demographic, clinical or sunlight associated variables assessed between both groups. The head and neck (36%) were the commonest sites of involvement (Figure 2).

**Figure 3.**
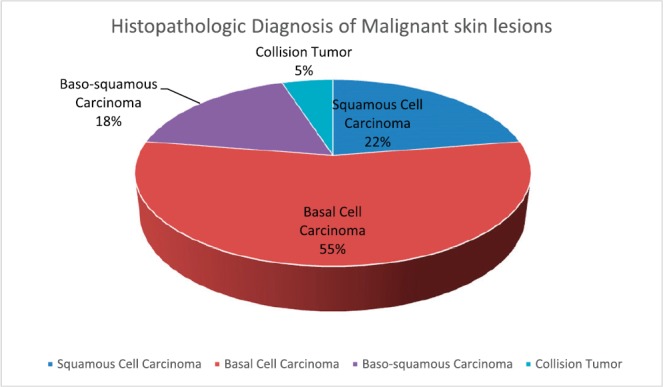
Histopathologic diagnosis of the cutaneous malignancies in the albinos.

**Figure 4.**
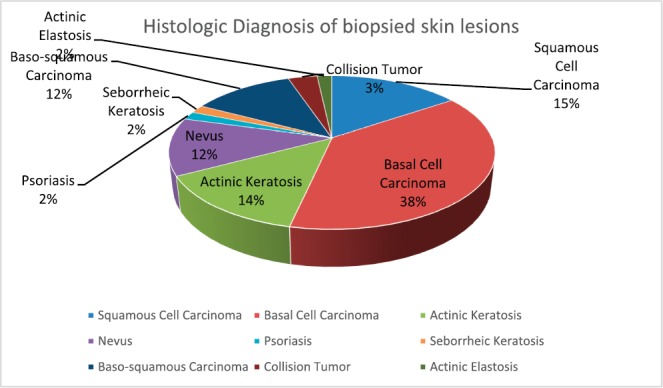
Histologic diagnosis of all of the biopsied skin lesions in the albinos.

Eighteen (60%) of the 30 albinos had malignant skin lesions comprising 9 (15%) SCCs, 22 (37.9%) BCCs, 7 (12%) basosquamous carcinoma and 2 (3.4%) collision tumour (BCC and SCC) (Figure 3). The rest of the biopsied lesions were intradermal and junctional nevi 7 (12%), actinic keratosis 9 (15%), solar elastosis 1 (1.7%) and psoriasis 1 (1.7%). (Figure 4).

## Discussion

Albinism is an established risk factor for keratinocyte skin cancers. Twenty percent (20%) of the patients examined had a form of cutaneous cancer. BCCs and SCCs were the commonest forms of skin cancers seen in this population of patients.

The greater frequency of BCCs in the study population highlights the importance of melanin in the protection of basal cells against UV mediated DNA damage. Consequently, the absence of pigmentation increases the chances of the development of BCCs. Several studies on skin photobiology have demonstrated that 70% of the total skin melanin content is in the basal layer of the skin [13]. BCCs are known to arise from mutated stem cells from the keratinocytes of the basal layer while SCCs arise from early progenitors which give rise to the suprabasal differentiated keratinocytes [13]. Therefore, it can be argued that reduced or absent melanin would cause more UV-mediated damage to the cells of the basal layer with more occurrence of BCCs in albinos rather than SCCs.

Albinos in our environment typically present to hospitals with advanced skin cancers [1]. Poor health-seeking behaviour as a result of ignorance, costs and aversion to surgical interventions are contributory. Unlike SCCs, BCCs are characteristically indolent, consequently, albinos with early BCCs are unlikely to present early to the hospital and are likely to be missed in hospital-based studies that rely solely on tissue samples submitted for histology. Furthermore, albinos are likely to opt for topical therapies, for example, the use of 5 fluorouracil for localized and locally infiltrative disease rather than come to the hospital for surgical excisions and biopsies, presenting to the hospitals only when the lesions become extensive. Community-based studies are thus likely to establish a diagnosis of early BCCs when compared to studies reporting skin cancer incidence from albinos presenting to the hospitals.

**Figure 5.**
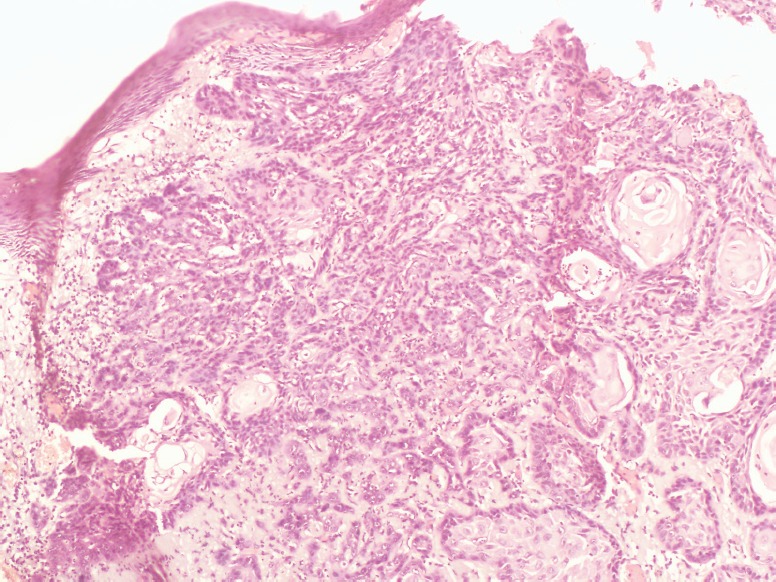
Photomicrograph showing basal cell carcinoma with actinic damage (Hematoxylin and Eosin, ×40).

BCC is a great mimic of several skin conditions like dermal nevi and can easily be missed. Dermoscopy has been found to be a useful non-invasive tool for the early detection of skin cancers and the exclusion of mimics in order to guide biopsies. The selection of suspected malignant lesions for biopsies with increased precision in albinos, who are likely to have many lesions in their sun-exposed areas, therefore, requires a careful and thorough search using both clinical and dermoscopic criteria for confirmation by histology. Studies employing Dermoscopy in the selection of lesions for biopsy will, therefore, be more representative of the pattern of keratinocyte skin cancers. The use of Dermoscopy (Figure 5) in this study may also explain the higher incidence of BCCs.

Again, aversion of albinos to invasive procedures like biopsies due to cost and poor acceptance of surgical procedures can lead to missed diagnosis and misclassification [18], especially if diagnosis rests on clinical criteria alone.

In addition, sunscreen use has been shown to inhibit the occurrence of SCCs and melanomas but not BCCs [23–26]. Free sunscreens are occasionally given to albinos in our environment. It is possible that increasing awareness of sun protection with increasingly more albinos using sunscreens may explain the higher frequency of BCCs when compared to SCCs in the current study.

Finally, the possibility of familial clustering of genetic loci responsible for BCCs may be another reason to consider for the high frequency of BCCs in our study population. This could be further explored by genetic studies among albinos in our environment.

This disproportionately higher frequency of BCCs among the albino population is consistent with several reports showing BCCs to be more common than SCCs among Caucasians [27, 28], fair-skinned normal pigmented Africans [6–10, 29] and among albinos [17, 30] than was earlier reported.

Although this differs from earlier studies which showed BCCs to be infrequent among African albinos [2, 15], the finding of SCC/BCC ratio of 1:2.3 further confirms that both BCCs and SCCs occur commonly in albinos comparable to the ratios reported in other studies in Africa which found a ratio of 1: 1.5 [17, 30], in lighter complexioned Africans [6–9] and in Caucasians [27, 28] and closely approximates the ratio reported by Kiprono *et al* [18] in Tanzania (Table 2) which found an SCC/BCC ratio of 1.2: 1 and Ademola *et al* [19] at Ibadan, Nigeria, with a ratio of 1:1.

Consistent with previous studies [3, 15, 16], no case of melanoma was reported, corroborating the reported rarity of melanomas in albinos.

The commonest site of the cutaneous malignancies was on the head and neck (36%) closely followed by the upper back (21%), the least common site being the abdomen (Figure 2) where SCC was found. This corroborates the findings in several studies [2, 3, 18] supporting the evidence that skin cancers in albinos occur commonly at sun-exposed sites.

This study is not without limitations. The smaller sample size of this study, when compared with larger retrospective studies, challenges the assertion that BCCs are more frequent in African albinos when compared with SCCs. Larger community-based research combined with dermoscopy and possibly genetic studies are, therefore, needed to validate this claim.

## Conclusion

Contrary to previous reports, it would appear that the pattern of cutaneous malignancies in albinos shows the same trend as that seen in Caucasians. We recommend that larger community studies combining dermoscopic criteria and histopathology be carried out in albinos to ascertain the prevalence of the different cutaneous malignancies in them.

## Funding

This authors received no specific funding for this research.

## Conflict of interest

The authors certify that they have no affiliations with or involvement in any organization or entity with any financial or non-financial interest in the subject matter or materials discussed in this manuscript.

## Figures and Tables

**Table 1.** Sociodemographic characteristics of the albinos with skin biopsies done.

	Malignant	Non-malignant	p-values
Gender, *N* (%)			
Male	9 (50.0)	5 (41.7)	0.654
Female	9 (50.0)	7 (58.3)	
Age, Median (IQR)	39 (33 – 47)	19 (10 – 33)	0.002
Hours in sun per week, Median (IQR)	14.9 (8.4 – 30.5)	9.0 (3.4 – 19.5)	0.316

**Table 2.** Comparison of reported cancer incidence among albinos.

Authors	Opara et al [2]	Asuquo et al [30]	Gilyoma et al (2012)	Kiprono et al [18]	Emadi et al [17]	Awe and Azeke [14]	Present study
Study design(total number of participant)	Retrospective Hospital based (20)	Retrospective Hospital based (9)	Retrospective Hospital based 64	Retrospective Hospital based (86)	Retrospective Hospital based 151	Retrospective Hospital based (22)	Cross sectional Community based (90)
SCC	84.2%	6(42.8%)	75%	53.7%	5.29	68.2%	15%
BCC	13.2%	7(50%)	23.4%	45.6%	7.94	22.7%	37.9%
Melanoma	-	1(7.1%)	1.6%	0.7%	-	9.1%	-
Basosquamous carcinoma	2.6%	-	-	-	-	-	12%
Collision tumor	-		-	-		-	3.4%
